# Vitamin D is associated with reduced risk of Sjögren’s syndrome: a Mendelian randomization study

**DOI:** 10.1093/rheumatology/kead356

**Published:** 2023-07-14

**Authors:** Sizheng Steven Zhao, Stephen Burgess

**Affiliations:** 1 Centre for Epidemiology Versus Arthritis, Division of Musculoskeletal and Dermatological Science, School of Biological Sciences, Faculty of Biological Medicine and Health, The University of Manchester, Manchester Academic Health Science Centre, Manchester, UK; 2 British Heart Foundation Cardiovascular Epidemiology Unit, Department of Public Health and Primary Care, University of Cambridge, Cambridge, UK; 3 Heart and Lung Research Institute, University of Cambridge, Cambridge UK; 4 Medical Research Council Biostatistics Unit, University of Cambridge, Cambridge, UK

Dear Editor,

Sjögren’s syndrome is an autoimmune disease characterised by lymphocytic infiltration of exocrine glands, but also systemic features such as arthritis, vasculitis, pulmonary and neurological dysfunction. Despite its impact on quality of life and physical function, no disease-modifying drugs have been approved. Some observational studies have linked vitamin D deficiency to increased Sjögren’s risk [1], but these findings are susceptible to confounding and reverse causation. Clinical trial evidence suggests that vitamin D supplementation reduces overall autoimmune disease risk [2], but whether this applies to Sjögren’s is not known. Mendelian randomization (MR) uses genetic variants as instrumental variables to estimate the effect of an exposure on an outcome that is typically more robust to these biases. Prior MR studies found no evidence for a link between vitamin D and Sjögren’s, but were limited by poor case definition and/or statistical power [3,4]. The aim of this MR study was to estimate the effect of genetically predicted vitamin D level on risk of primary Sjögren’s syndrome.

Genetic data for primary Sjögren’s syndrome was taken from the largest genome-wide association study (GWAS) to date of 6,098 cases (defined by American-European Consensus Group criteria) and 34,928 controls [5]. We used data from a GWAS of 25 hydroxyvitamin D (25OHD) concentration in 417,580 individuals of European ancestry [6]; associations were adjusted for age, sex, assessment month, assessment centre, first 40 principal components, genotyping batch and supplement intake. To minimize risk of collider bias, primary analysis used genetic associations unadjusted for BMI. Because some lead variants for 25OHD are associated with body composition, we also used data that conditioned on BMI as sensitivity analysis. To minimize potential bias due to horizontal pleiotropy, we selected variants (p<5x10^-8^, r^2^<0.1 and present in the Sjögren’s GWAS) from four gene regions previously shown to be strongly associated with 25(OH)D and implicated in the transport, metabolism, and synthesis of vitamin D – *GC*, *DHCR7*, *CYP2R1*, and *CYP24A1* [7]. Our instrument selection differed from prior one-sample (applying step-wise conditional selection to individual-level data [3,7]) and two-sample MR analyses (using variants across the genome [4]). We used the inverse-variance weighted (IVW) method and pleiotropy robust sensitivity analyses, i.e., MR Egger and weighted median/mode methods. IVW and Egger methods additionally accounted for weak correlation between variants. MR estimates were scaled to per standard deviation (approximately 21nmol/L) increase in 25OHD.

Fifteen variants were selected to instrument 25OHD (supplementary table S1) that together explained 3.2% of the variance in 25OHD concentration, corresponding to a mean F statistic of 931. Genetically predicted 25OHD was associated with reduced risk of Sjögren’s syndrome (OR 0.72 perstandard deviation decrease in 25OHD; 95%CI 0.57, 0.90; p=0.004). Estimates were directionally concordant in pleiotropy robust sensitivity analyses and analysis accounting for BMI (supplementary figure S1) and appear to be mostly driven by rs11023374 in the *CYP2R1* gene (supplementary figure S2).

This study is the first to provide genetic evidence that higher circulating vitamin D levels may reduce risk of primary Sjögren’s syndrome. Our analyses differ from earlier MR studies of vitamin D that were limited by small number of poorly defined cases, which may explain their null findings [3,4]. MR studies have failed to replicate most traditional observational associations between vitamin D and autoimmune diseases (with the exception of MS [8]) likely due to confounding and/or reverse causation in traditional designs. The VITAL trial supported a role of vitamin D for reducing autoimmune diseases but was not powered to interrogate individual diseases [2]. The shared immune mechanisms (abnormal B and T cell response to autoantigens) between MS and Sjögren’s may provide mechanistic insight into the potential effect of vitamin D on autoimmunity [3].

One limitation is that risk factors for disease incidence, which were the focus of this study, may not be relevant to progression of established disease. The key assumption of no pleiotropic pathways cannot be empirically verified. However, we tried to mitigate this by selecting instruments from genes relevant to vitamin D biology. Sensitivity analyses were also consistent with the main results. In summary, we found genetic evidence to support a causal link between vitamin D and Sjögren’s syndrome. Vitamin D supplementation could represent a relatively safe and inexpensive therapeutic option, but these findings need to be confirmed in randomized controlled trials. These results have implications for potential disease prevention strategies and the interpretation and design of future trials.

## Supplementary Material

Supplementary material

## Data Availability

Summary statistics for Sjögren’s syndrome is accessible in the Databases of Genotypes and Phenotypes (dbGaP) under accession number phs002723.v1.p1. Summary statistics for vitamin D can be accessed via https://cnsgenomics.com/content/data.
