# Supplementary material for: Vitamin D is associated with reduced risk of Sjögren’s syndrome: a Mendelian randomization study
Source: Rheumatology (Oxford). Author manuscript; Available in PMC 2024 Feb 5. (PMC10836976; doi:10.1093/rheumatology/kead356)

**Supplementary materials for**

**Vitamin D is associated with reduced risk of Sjögren’s syndrome: a Mendelian randomization study**

Sizheng Steven Zhao, MD, PhD^1^, Stephen Burgess, PhD^2,3,4^

1 Centre for Epidemiology Versus Arthritis, Division of Musculoskeletal and Dermatological Science, School of Biological Sciences, Faculty of Biological Medicine and Health, The University of Manchester, Manchester Academic Health Science Centre, Manchester, UK

2 British Heart Foundation Cardiovascular Epidemiology Unit, Department of Public Health and Primary Care, University of Cambridge, Cambridge, UK

3 Heart and Lung Research Institute, University of Cambridge, Cambridge UK

4 Medical Research Council Biostatistics Unit, University of Cambridge, Cambridge, UK

Contents

[Table S1. Variants used to instrument vitamin D. 2](#_Toc138236931)

[Figure S1. Mendelian randomization estimates from primary and sensitivity analyses of vitamin D and Sjogren’s syndrome. 3](#_Toc138236932)

[Figure S2. Single variant estimates (left) and leave-one-out estimates (right) showing importance of rs11023374. 4](#_Toc138236933)

# Table S1. Variants used to instrument vitamin D.

| SNP | Chromosome | Position | Effect allele | Other allele | Vitamin D | | Vitamin D  conditioned on BMI | | Sjogren’s syndrome | |
| --- | --- | --- | --- | --- | --- | --- | --- | --- | --- | --- |
|  |  |  |  |  | beta | se | beta | se | beta | se |
| rs705117 | 4 | 72608115 | C | T | 0.033 | 0.003 | 0.034 | 0.003 | 0.029 | 0.041 |
| rs2282680 | 4 | 72608364 | C | T | 0.192 | 0.002 | 0.192 | 0.002 | -0.064 | 0.032 |
| rs79444700 | 4 | 72620557 | T | C | -0.084 | 0.005 | -0.083 | 0.005 | 0.035 | 0.079 |
| rs115103883 | 4 | 72621395 | T | C | -0.094 | 0.007 | -0.095 | 0.007 | 0.096 | 0.109 |
| rs222029 | 4 | 72644962 | G | A | 0.053 | 0.003 | 0.053 | 0.003 | 0.001 | 0.040 |
| rs74706296 | 4 | 72664972 | C | T | -0.041 | 0.003 | -0.041 | 0.003 | 0.028 | 0.048 |
| rs76781122 | 4 | 72669661 | C | A | -0.047 | 0.007 | -0.049 | 0.006 | -0.089 | 0.093 |
| rs117913124 | 11 | 14900931 | G | A | 0.377 | 0.006 | 0.377 | 0.006 | 0.060 | 0.094 |
| rs11023374 | 11 | 14903636 | T | C | 0.070 | 0.002 | 0.071 | 0.002 | -0.125 | 0.032 |
| rs12419334 | 11 | 71139472 | T | C | -0.106 | 0.002 | -0.106 | 0.002 | 0.036 | 0.032 |
| rs77073138 | 11 | 71140784 | T | C | 0.105 | 0.008 | 0.105 | 0.008 | -0.100 | 0.095 |
| rs1790334 | 11 | 71155153 | A | G | -0.096 | 0.005 | -0.097 | 0.005 | 0.027 | 0.068 |
| rs1570669 | 20 | 52774427 | A | G | -0.022 | 0.002 | -0.023 | 0.002 | 0.013 | 0.031 |
| rs3787557 | 20 | 52783135 | T | C | -0.021 | 0.003 | -0.021 | 0.003 | 0.007 | 0.043 |
| rs13038432 | 20 | 52787302 | A | G | 0.039 | 0.004 | 0.039 | 0.004 | 0.048 | 0.055 |

# Figure S1. Mendelian randomization estimates from primary and sensitivity analyses of vitamin D and Sjogren’s syndrome.


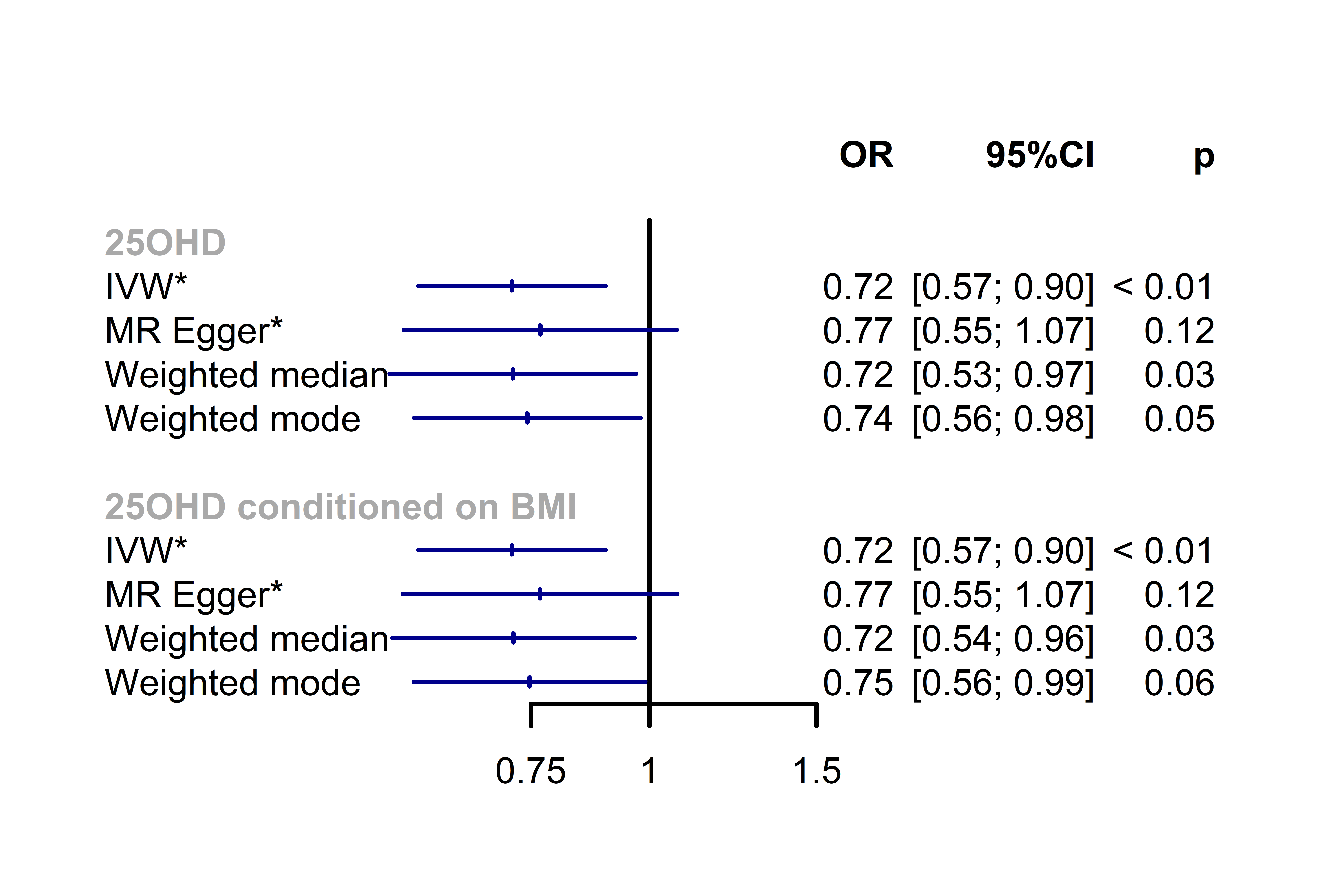


*analyses accounted for weak correlation between instruments. 25OHD, 25 hydroxyvitamin D; IVW, inverse variance weighted method. OR, odds ratio.

# Figure S2. Single variant estimates (left) and leave-one-out estimates (right) showing importance of rs11023374.


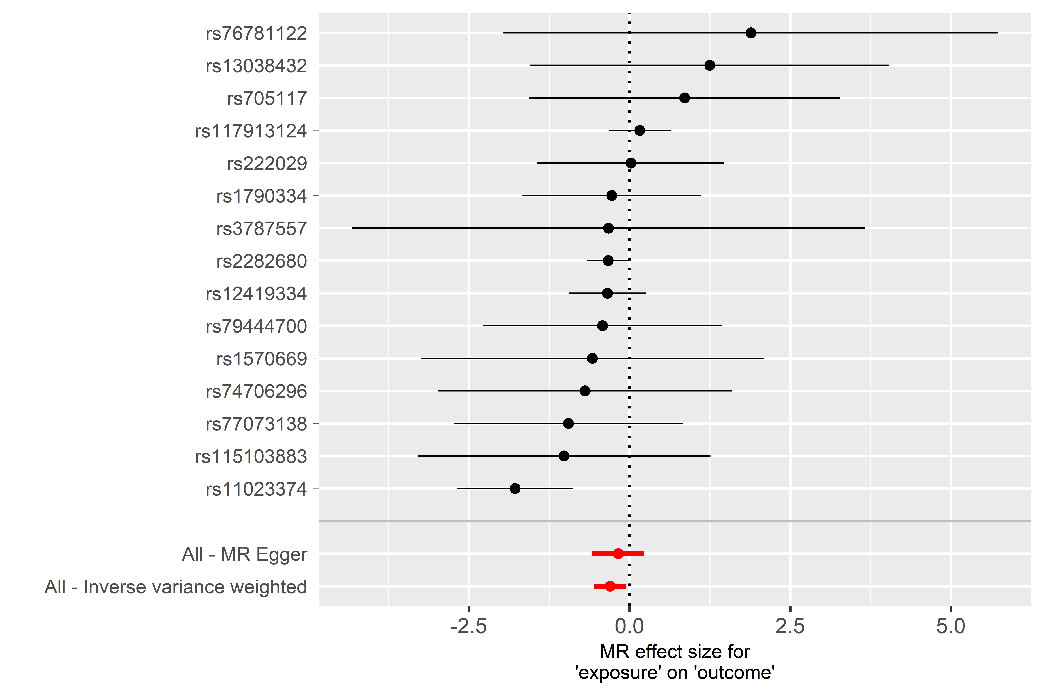

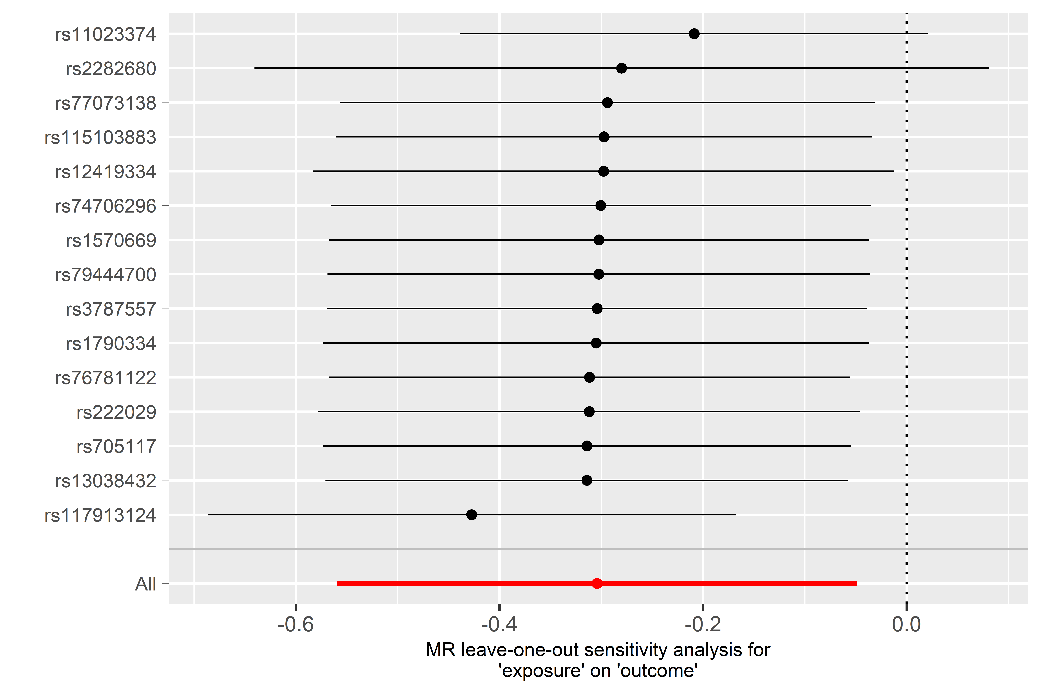

Supplement: Supplementary material [file EMS180730-supplement-Supplementary_material.docx]
